# Cost-utility of long-term exercise therapy in people with axial spondyloarthritis and severe functional limitations: a randomized, controlled trial

**DOI:** 10.1007/s00296-026-06160-x

**Published:** 2026-06-05

**Authors:** Maria. A. T. van Wissen, Salima F. E. van Weely, Cornelia. H. M. van den Ende, Theodora P. M. Vliet Vlieland, Max M. H. Teuwen, Wilfred. F. Peter, Eline A. M. Mahler, Dirkjan van Schaardenburg, Floris van Gaalen, Johanna P. L. Spoorenberg, Astrid M. van Tubergen, Maaike G. J. Gademan, Wilbert B. van den Hout

**Affiliations:** 1 Department of Orthopaedics, Rehabilitation and Physical Therapy, Leiden University Medical Center, Albinusdreef 2, P.O.Box 9600, Leiden, 2300 RC The Netherlands; 2 Institute of Allied Health Professions, HU University of Applied Sciences, Utrecht, Netherlands; 3 Department of Research, Sint Maartenskliniek, Nijmegen, The Netherlands; 4 Department of Rheumatology, Radboud UMC, Nijmegen, The Netherlands; 5 Center for Rehabilitation and Rheumatology, Reade, Amsterdam, The Netherlands; 6 Department of Rheumatology, Sint Maartenskliniek, Nijmegen, The Netherlands; 7 Department of Rheumatology, Leiden University Medical Center, Leiden, The Netherlands; 8 Department of Rheumatology, University Medical Center Groningen, Groningen, The Netherlands; 9 Department of Rheumatology, Maastricht University Medical Center, Maastricht, The Netherlands; 10 The Netherlands and Care and Public Health Research Institute (CAPHRI), Maastricht University, Maastricht, The Netherlands; 11 Department of Clinical epidemiology, Leiden University Medical Center, Leiden, The Netherlands; 12 Department of Biomedical Data Sciences, Leiden University Medical Center, Leiden, The Netherlands

**Keywords:** “Axial Spondyloarthritis"[Mesh], “Exercise Therapy"[Mesh], “Cost-Benefit Analysis"[Mesh], “Quality of life” [Mesh]

## Abstract

**Abstract:**

To evaluate the cost-utility of long-term, personalized, exercise therapy as compared to usual care in people with axial spondyloarthritis (axSpA) and severe functional limitations. A comprehensive economic evaluation was conducted from a societal perspective alongside a randomized controlled trial involving 214 participants (110 individuals assigned to the intervention group and 104 to the usual care group), with a one-year follow-up. Cost assessments encompassed both medical and non-medical costs recorded by participants and healthcare providers. Quality Adjusted Life Years (QALYs) were calculated using the EuroQol-5 Dimensions -5 Levels (EQ-5D-5L) and EuroQol Visual Analogue Scale (EQ-VAS). Costs and QALY differences were analysed using standard unequal variance t-tests according to the intention-to-treat principle and cost-effectiveness acceptability curves. In the intervention group, 93% of the participants used the intervention with an average of 41 (standard deviation, SD 15) sessions, with the mean direct costs of the intervention being €1515 (SD 724) per participant. The total mean physiotherapy costs were €1967 (SD 801) in the intervention and €514 (SD 792) in the control group, respectively. Although not statistically significant, the total societal costs also favoured the usual care group after 52-weeks, showing a difference of €657 (95% confidence interval (CI) €-3748 to €5060). QALYs were slightly and non-significantly favouring the intervention group, with a difference of 0.02 based on the EQ-5D-5 L (95% CI − 0.04 to 0.09) and 0.00 according to the EQ-VAS (95% CI -0.04 to 0.04). At a willingness-to-pay threshold of €50,000 per QALY, the intervention had a 57% likelihood of being considered the cost-effective strategy. The higher intervention costs of the long-term exercise therapy intervention were negated by savings on other healthcare and non-healthcare costs and by improved QALYs. As a result, we found no clear economic preference: long-term exercise therapy need not be withheld for economic reasons from people with axSpA and severe functional limitations.

**Registration number:**

Netherlands Trial Register NL-OMON52399, included in the International Clinical Trial Registry Platform (ICTRP) (https://trialsearch.who.int/Trial2.aspx? TrialID=NL-OMON52399).

## Introduction

Axial SpondyloArthritis (axSpA) is a chronic rheumatic condition characterized by progressive inflammation of the spine and sacroiliac joints, which may lead to spinal ankylosis [1, 2]. The disease affects approximately 0.3%-1.4% of the global population and substantially impacts physical functioning and quality of life [1–4]. The management of axSpA involves pharmacological interventions and non-pharmacological care, with patient education and exercise therapy being fundamental components [5, 6]. Several systematic reviews [7–9] and a recent umbrella review showed that exercise therapy in axSpA has various beneficial effects, including favourable effects on pain, disease activity, axial mobility, and functional ability [7–10]. However, most studies involved highly selected patients, excluding those with e.g., comorbidities, severe ankylosis or joint arthroplasties. Recently, the effectiveness of exercise therapy has been evaluated in people with axSpA and severe functional disability due to e.g. comorbidities, ankylosis, or involvement of peripheral joints [11]. In this randomized controlled trial (RCT, the Longstanding EXercise therapy in axial SPondyloArthritis (L-EXSPA) study)), the effectiveness of a long-term (52 weeks), personalized exercise intervention as compared to usual care was demonstrated for functional ability and quality of life [12]. However, its economic benefit remains unestablished.

Economic analyses regarding exercise therapy in axSpA are scarce. One economic analysis was done alongside an RCT comparing group exercise therapy with unsupervised individual home exercise therapy for 9 months in people with ankylosing spondylitis (AS) [13]. This study found that the benefits (improvement of mobility and fitness) of group therapy costed $531/year, but reduced medical costs with $122/year. Another study analysed the cost-effectiveness of exercise therapy as part of 3-week spa-exercise treatment program for people with AS [14]. This study concluded that the spa-exercise therapy in addition to standard treatment (including medication and weekly group exercise therapy) was more effective and showed favourable cost-effectiveness. The between-group difference on quality-adjusted life years (QALYs) was significantly in favour of the spa-exercise treatment group. However, these were conducted years ago, before the introduction of biological Disease Modifying Antirheumatic Drugs for axSpA, which may affect their relevance to current medical practice. Moreover, these studies did not make a comparison between exercise therapy and usual care and they focused only on people with AS, limiting the generalizability of their findings to non-radiographic (nr-) axSpA.

The economic analysis that we recently did alongside the parallel RCT on the effectiveness of longstanding exercise therapy in patients with rheumatoid arthritis (RA) [11, 15] may thus be more relevant. In that study [15], the costs were higher in the intervention group, with a small difference of €180 [95% confidence interval (CI) €−4493 to €4852], whereas the QALYs were non-significantly in favor of the intervention group, by 0.02 according to the EuroQol 5 Dimensions 5 Levels (EQ-5D-5 L) (95% CI − 0.05 to 0.09) and by 0.04 according to the EQ-Visual Analog Scale (VAS) (95% CI 0.00 to 0.08). Another relevant study concerns an RCT on the effectiveness of personalized, goal-oriented exercise therapy for elderly people with mobility problems, showing that the physical therapy treatment (motivational interviewing, physical examination, individualized goal setting, and coaching and advice ons self-management regarding physical activity) resulted in significant cost savings compared with usual care [16]. Despite their similarities, both studies were carried out in different populations and their results may thus not be generalizable to people with axSpA with severe functional limitations.

Given the knowledge gap regarding the cost-effectiveness of longstanding exercise therapy in people with axSpA and severe functional disability, the aim of the present study was to determine its cost-utility in comparison with usual care.

## Patients and methods

### Study design

This economic evaluation was conducted alongside an assessor-blinded RCT comparing the effectiveness of a long-term (52 weeks) personalized exercise therapy intervention to usual care (the L-EXSPA study). The study was coordinated by the Leiden University Medical Center and executed between October 2019 and December 2023. Details of the study design were published earlier [11, 12]. The anticipated sample size of 215 participants was based on clinical outcomes [11]. The cost-utility analysis was done according to Dutch economic evaluation guidelines [17, 18], from the societal perspective and with a time horizon of 52 weeks. The study was approved by the Medical Ethical Review Board Leiden-Delft-Den Haag on July 11, 2019, and all participants provided written informed consent. The study was registered in the Overview of Medical Research in the Netherlands (OMON) (https://www.onderzoekmetmensen.nl/en) with number NL-OMON52399. OMON is an official data provider to the International Clinical Trial Registry Platform (ICTRP) of the World Health Organization. Reporting of the study was done according to the Consolidated Health Economic Evaluation Reporting Standards (CHEERS) 2022 checklist and CONSORT [19, 20].

## Study population

Eligible individuals were adults (aged ≥ 18 years) with a clinical diagnosis of axSpA as confirmed by their rheumatologist. These individuals experienced self-perceived severe limitations in basic daily activities related to self-care (such as dressing and washing), transfers (including getting in and out of bed, rising from a chair or using the toilet), and/or mobility indoors or outdoors. The limitations were directly or indirectly linked to their axSpA, e.g., being caused by persisting or progressive disease activity despite optimal medical treatment and/or severe ankylosis and/or deformities and/or severe comorbidities (e.g., pulmonary or cardiovascular disease, obesity). Additionally, their functional limitations were unlikely to improve or be resolved with a brief exercise therapy intervention. Individuals who had undergone physical therapy in the past three months, or those who were in need for admission to a hospital or rehabilitation centre, were excluded from the study. If a potential participant was using physical therapy but met the other eligibility criteria, he/she could still participate if physical therapy was stopped for a minimum of three months.

## Intervention and control conditions

The intervention comprised a 52-week individualized and supervised active exercise program. Treatment was delivered by a trained primary care physical therapist (PT) located near each participant, either at the PT practice or in the participant’s home environment. All PTs completed mandatory training through either in-person sessions or an e-learning platform. The intervention followed a standardized treatment protocol that included an initial assessment, formulation of treatment goals, and delivery of active therapy, consisting of exercise therapy, physical activity counseling, and patient education. Treatment was tailored to individual participants on the basis of ongoing monitoring and periodic evaluations. PTs were advised to provide two sessions per week during the initial 12 weeks, followed by a reduction to one session weekly thereafter. In addition, up to 14 supplementary sessions could be offered depending on participants’ individual needs, functional limitations, and progress toward treatment goals.

Participants allocated to the usual care group received standard care as determined jointly by their treating clinician(s) and themselves. Usual care could include standard physical therapy through referral or direct access, provided that the PT was not involved in treating participants assigned to the intervention group. After completion of the 52-week study period, participants in the usual care group were offered access to the intervention.

## Assessments

### Sociodemographic and disease characteristics

Following enrolment, participants completed a questionnaire on sociodemographic and health-related information. Data collected included age (years), sex (male/female/other), height (m), and body weight (kg), which were used to calculate body mass index (BMI). Additional variables included living status (single-person household: yes/no), educational attainment (low/intermediate: primary or secondary (vocational) education; high: Bachelor’s or Master’s degree from a university or university of applied sciences), employment status for participants aged ≤ 66 years (paid employment: yes/no), and supplementary health insurance coverage (yes/no). Clinical and lifestyle information included self-reported symptom duration (years), smoking history (current or former smoker: yes/no), and the presence of 19 predefined comorbidities assessed using a questionnaire developed by Statistics Netherlands [21]. Physical functioning was evaluated using the Bath Ankylosing Spondylitis Functional Index (BASFI) [22, 23].

In addition, treating rheumatologists provided clinical characteristics, including classification as radiographic or non-radiographic axSpA, the Bath Ankylosing Spondylitis Disease Activity Index (BASDAI) score, and time since diagnosis in years.

### Utility measures and QALYs

Health utility values, reflecting the valuation of quality of life on a scale from 0 (equivalent to death) to 1 (perfect health), were assessed at baseline and after 12, 26, and 52 weeks using two approaches. General health status was measured with the EuroQol-5 Dimensions-5 Levels (EQ-5D-L), which evaluates mobility, self-care, usual activities, pain/discomfort, and anxiety/depression [24]. Subsequently, the Dutch utility index was calculated from the EQ-5D-5L responses [25]. Participants also rated their overall health status using the EuroQol Visual Analog Scale (EQ-VAS), ranging from 0 (worst imaginable health state) to 100 (best imaginable health state). EQ-VAS scores were converted into utility values using the transformation formula: 1–(1–EQ-VAS/100)^1.61^ [26]. One-year QALYs were estimated by calculating the area under the curve for each utility measure across the follow-up period. QALYs are widely used in economic evaluations to support healthcare resource allocation decisions [27].

### Costs

Societal costs over the 1-year follow-up period were estimated using 2023 price levels without discounting. At 12, 26, and 52 weeks, participants completed questionnaires addressing healthcare utilization (including physical therapy), domestic assistance, informal care, paid employment hours, absenteeism, presenteeism, and reduced unpaid productivity. These questionnaires were adapted from the Institute for Medical Technology Assessment (iMTA) Medical Consumption Questionnaire (iMCQ) and Productivity Cost Questionnaire (iPCQ) [28, 29].

For participants assigned to the intervention group, physical therapists additionally documented the number of treatment sessions delivered. These PT-registered sessions were used to calculate the costs of the long-term exercise intervention. When participants in the intervention group reported more physical therapy sessions than their treating PT, the difference was designated as a “discrepancy between patient-reported and PT-registered physical therapy” and counted as costs. Thus, the maximum of the patient-reported and PT-reported number of sessions was the basis for the total costs of physical therapy in the intervention group. In the usual care group, the only source for the use of physical therapy was “patient-reported physical therapy”.

Whenever available, Dutch standard reference prices were applied to value healthcare utilization [30, 31]; otherwise, market-based prices were used. Travel expenses were estimated from the reported number of healthcare visits in combination with national average travel distances and transportation methods [18]. Costs related to domestic assistance, informal caregiving, and unpaid productivity losses were valued at €17 per hour [18]. Productivity losses from paid work were calculated using the friction cost approach, applying a valuation of €42 per hour and a maximum friction period of three months [18, 30, 31].

### Statistical analyses

Data were analysed using SPSS, IBM Corp (Released 2017, IBM SPSS Statistics for Windows, Version 25.0. Armonk, NY: IBM Corp). The cost-utility analysis was similar to those employed in the parallel RCT on longstanding exercise in patients with RA [15] and in line with the registered study protocol [11]. Statistical comparisons were performed using standard unequal variance t-tests according to the intention-to-treat principle. Multiple imputation with MICE (100 imputed data sets) was used to address missing data and to preserve statistical power and reduce bias [32]. The imputation model included randomization, sex, age, EQ-5D-5L scores at baseline, 12, 26 and 52 weeks, and the Patient-Specific Complaints activity ranked 1 at baseline and 52 weeks (the study’s primary outcome). For our sample size, research has shown that parametric methods are robust enough and they are more compatible with multiple imputation than bootstrapping [33].

Cost-effectiveness acceptability curves were generated to illustrate the likelihood that long-term exercise therapy would be considered cost-effective relative to usual care across varying levels of societal willingness to pay (WTP) for an additional QALY. These curves were calculated as the one-sided p-value for the differences in Net Benefit = WTP ⋅ QALY - Costs between the participants in the intervention and the usual care group [34]. In the Netherlands, the relevant WTP threshold is €20,000, €50,000, or €80,000 per QALY, depending on disease burden [35]. For the present study population, a threshold of €50,000 per QALY was considered the most suitable reference value.

In the primary economic analysis, total societal costs were assessed in relation to QALYs derived from the utility index scores for the EQ-5D-5L. Sensitivity analyses were subsequently conducted using an alternative utility measure (EQ-VAS), different cost measures (costs from a medical perspective and only intervention costs) and by adjusting for relevant baseline imbalances (using linear regression analysis instead of t-tests). Incremental cost-effectiveness ratios (ICERs) were calculated as the difference in costs divided by the difference in QALYs, with confidence intervals calculated as those WTP values for which the net benefit was statistically significant.

## Results

Originally, 215 participants were included and randomized, but in one patient the diagnosis of axSpA was eventually not confirmed, and the patient was excluded from the study. Therefore, the economic evaluation encompassed a total of 214 participants, with 110 individuals assigned to the intervention group and 104 to the usual care group (Table 1). The participants’ mean ages were 51.9 (SD 11.7) years and 52.4 (SD 12.1) years in the intervention and usual care groups, respectively. Fifty-six out of 110 (50.9%) participants in the intervention group were female and 49/104 (47.1%) participants in the usual care group. The proportions of participants with one or more joint arthroplasties were relatively similar (11/110 (10%) and 15/104 (14%), respectively). Forty-six out of 106 (43.4%) participants in the intervention group had five or more comorbidities, compared to 38/101 (37.6%) participants in the usual care group. With respect to disease activity, 44/64 (69%) participants of the intervention group had a BASDAI score of ≥ 4, compared to 46/70 (66%) participants in the usual care group. The BASFI scores were 6.0 (SD 2.1) and 5.9 (SD 1.8) in the intervention and usual care groups, respectively. These results reflect that the study population comprised individuals with severe functional disability and that the groups seemed relatively comparable with regard to the assessed patient characteristics. Regarding health resource utilization and productivity measurements, 9% had missing data, while utility measurements had 8% missing data.

**Table 1:** Baseline demographic and health characteristics of people with axSpA and severe functional limitations participating in the L-EXSPA study

	Intervention group (*N* = 110)	Usual care group (*N* = 104)
Female, *N* (%)	56 (50.9)	49 (47.1)
Age in years, mean (SD)	51.9 (11.7)	52.4 (12.1)
BMI (kg/m2), mean (SD)	28.0 (5.1) (*n* = 107)	28.1 (5.5) (*n* = 104)
Single-person household, N (%)	26 (23.9) (*n* = 109)	22 (21.2) (*n* = 104)
Higher education, N (%)	47 (43.1) (*n* = 109)	31 (29.8) (*n* = 104)
Work status, N (%)		
≤ 66 years old, N (%)	97 (88.2)	90 (86.5)
Paid job, N (%)	33 (34.0)	34 (37.8)
No job, health problems,* N (%)*	22 (22.7)	16 (17.8)
No job,other reasons, N (%)	42 (43.3)	40 (44.4)
Health insurance with additional coverage, N (%)	94 (87.0) (*n* = 108)	83 (81.4) (*n* = 102)
Self-reported duration of complaints (years), mean (SD)	23.5 (12.9) (*n* = 107)	24.6 (14.9) (*n* = 102)
Years since diagnosis , Mean (SD)	14.1 (11.3) (*n* = 97)	16.1 (14.8) (*n* = 91)
Radiographic axial spondyloarthritis, N (%)	74 (80) (*n* = 93)	79 (87) (*n* = 91)
BASDAI, Mean (SD)	4.9 (2.1) (*n* = 64)	5.0 (1.6) (*n* = 70)
BASDAI > 4, N (%)	44 (69) (*n* = 64)	46 (66) (*N* = 70)
BASFI, Mean (SD) (0–10)	6.0 (2.1) (*n* = 105)	5.9 (1.8) (*n* = 99)
Smoking status: Ever smoked, N (%)	64 (59) (*n* = 108)	67 (66) (*n* = 102)
Number of comorbidities, N (%)	(*n* = 106)	(*n* = 101)
0	4 (3.8)	11 (10.9)
1–2	23 (21.7)	24 (23.8)
3–4	33 (31.1)	28 (27.7)
≥ 5	46 (43.4)	38 (37.6)
Joint replacement surgeries (≥ 1), N (%)	11 (10)	15 (14)

Abbreviations and explanations: BMI Body Mass Index; Higher education, Bachelor or Master degree at a university or university of applied sciences; BASDAI. Bath Ankylosing Spondylitis Disease Activity Index; BASFI, Bath Ankylosing Spondylitis Functional Index

### Utilities and clinical outcome

**Fig. 1:**
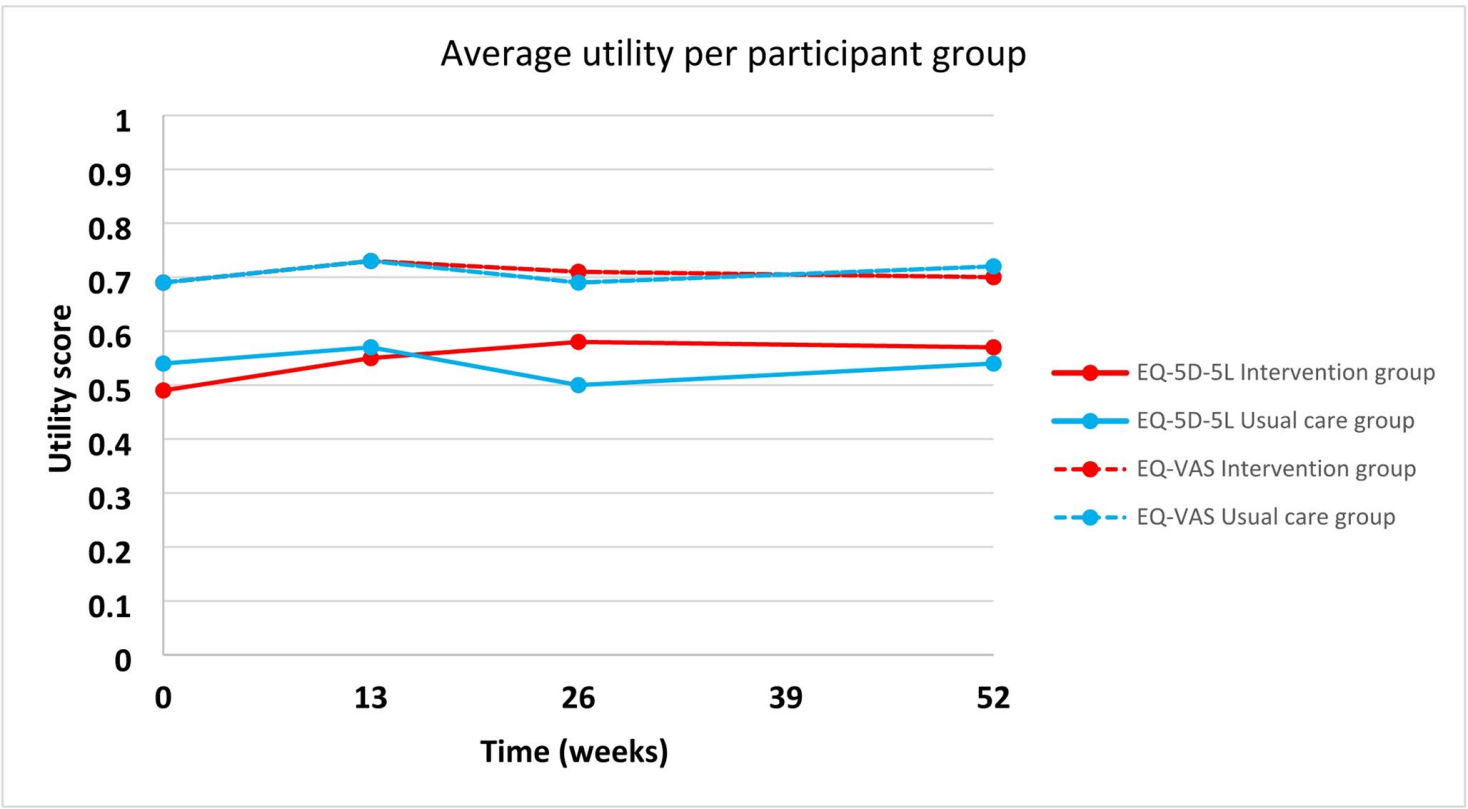
Mean utility, by time and treatment group. EQ-5D-5L, EuroQol-5Dimensions-5Levels; EQ-VAS, EuroQol Visual Analogue Scale

Table 2 provides an overview of the mean EQ-5D-5L and EQ-VAS scores at the various time points. After one year, the average health valuation for both utility measures favoured the intervention group over the usual care group, as illustrated in Fig. 1. Notably, only the EQ-5D-5L score at 26 weeks showed a statistically significant difference (0.08, 95% CI 0.00 to 0.15, *p* = 0.04).

**Table 2:** Average Utility scores and QALYs in the intervention and usual care groups

EQ-5D-5L utility score	Intervention group (*n* = 110) mean (SD)	Usual care group (*n* = 104) mean (SD)	MD*	95%CI	*P*†
Baseline	0.49 (0.27)	0.54 (0.24)	-0.05	-0.12–0.02	0.13
12 weeks	0.55 (0.26)	0.57 (0.26)	-0.02	-0.10–0.05	0.51
26 weeks	0.58 (0.25)	0.50 (0.28)	0.08	0.00–0.15	0.04
52 weeks	0.57 (0.28)	0.54 (0.24)	0.04	-0.04–0.11	0.33
QALY EQ-5D-5L	0.56 (0.23)	0.53 (0.22)	0.02	-0.04–0.09	0.43
EQ-VAS utility score					
Baseline	0.69 (0.20)	0.69 (0.19)	0.00	-0.05–0.06	0.90
12 weeks	0.73 (0.17)	0.73 (0.18)	-0.01	-0.05–0.04	0.81
26 weeks	0.71 (0.19)	0.69 (0.19)	0.02	-0.03–0.07	0.43
52 weeks	0.70 (0.20)	0.72 (0.16)	-0.02	-0.07–0.03	0.49
QALY EQ-VAS	0.71 (0.15)	0.71 (0.14)	0.00	-0.04–0.04	0.90

Mean values are shown for each assessment point (baseline; 12 weeks; 26 weeks; 52 weeks).

*MD = mean difference between intervention and usual care group.

†The P-values and 95% Confidence Intervals (CI) for the differences between intervention and usual care group.

Abbreviations: QALY, Quality Adjusted Life Years; EQ-5D-L, EuroQol-5Dimensions-5Levels; EQ-VAS, EuroQol Visual Analog Scale with power transformation

### Costs of long-term exercise therapy

Table 3 shows the costs per patient in both the intervention and usual care groups. Of the participants in the intervention group, 102/110 (93%) actively participated in the long-term personalized exercise therapy intervention. Across the entire intervention group, the mean number of sessions was 38, and for those who utilized the intervention, it was 41. The mean (SD) total costs of the longstanding exercise therapy intervention in the intervention group were estimated at €1515 (724) per patient. Fifty-nine out of 110 (54%) participants in the intervention group reported therapy sessions above the study sessions reported by their PT. The mean (SD) costs of these additional sessions were €452 (646). Due to a logistical error, two participants (2%) in the usual care group were inadvertently granted access to the intervention before 52 weeks. They started one week too early with the intervention, which led to a mean (SD) intervention cost in the usual care group of €1 (6). In the usual care group, 67% of the participants reported the use of physical therapy, with the mean (SD) costs per patient being €513 (784). The total costs of physical therapy were €1967 (801) in the intervention group and €514 (792) in the usual care group, with a difference of €1454 (95% CI €1231 to €1676, *p* < 0.001).

**Table 3:** Mean 1-year medical and non-medical costs per participant, by group

	Intervention group (*n* = 110)		Usual care group (*n* = 104)		Difference in costs between groups		
	Volume	Costs (€)	Volume	Costs (€)		95% CI	*P*
Physiotherapy, visits							
Intervention, at centre^a^	93%^#^ 41	1515	2%^#^ 1	1	1515	1379 to 1650	< 0.001
Intervention, at home^a^	0%^#^ 0	0	0%^#^ -	0			
Total intervention physiotherapy^a^**(SD)**	93%^#^ 41	1515 (724)	2%^#^ 1	1 (6)	1515	1379 to 1650	< 0.001
Patient-reported physiotherapy^a^			67%^#^ 19	513	-513		
Discrepancy in patient-reported and physical therapist-registered physiotherapy^a^	54%^#^ 21	452			452		
Total physiotherapy (SD)	99%^#^ 50	1967 (801)	67%^#^ 19	514 (792)	1454	1231 to 1676	< 0.001
General practitioner	4.8	170	5.0	177	-6	-52 to 40	0.79
Specialists							
Rheumatologist, visits	3.1	345	3.4	383	-37	-170 to 95	0.58
Orthopedic surgeon, visits	0.3	39	0.4	41	-2	-41 to 36	0.90
Internist, visits	0.6	69	0.3	37	32	-13 to 77	0.17
Cardiologist, visits	0.4	42	0.4	42	0	-27 to 26	0.98
Other, visits^b^	2.4	269	2.3	258	11	-104 to 125	0.85
Other healthcare providers							
Rheumatology nurse, visits	1.0	20	0.7	15	5	-4 to 14	0.27
Podiatrist, visits	0.2	9	0.3	14	-4	-15 to 6	0.41
Occupational therapist, visits	0.5	20	0.6	24	-4	-31 to 24	0.80
Dietitian, visits	0.8	27	0.2	8	20	5 to 35	0.01
Social worker, visits	0.04	3	0.05	4	-1	-7 to 6	0.84
Other, visits^c^	1.6	84	0.8	47	37	-56 to 131	0.43
Day treatment							
Hospital, visits	0.7	203	0.7	203	1	-152 to 153	0.99
Rehabilitation center, visits	1.8	1119	1.2	770	348	-967 to 1664	0.60
Psychotherapeutic institution, visits	1.6	351	1.3	305	46	-347 to 439	0.82
Inpatient hospitalization							
Hospital, days	0.8	489	1.1	653	-164	-608 to 279	0.47
Rehabilitation center, days	1.1	604	2.4	1233	-629	-2000 to 741	0.37
Psychotherapeutic institution, days	0	0.0	0	0.0	-	-	-
Home care, hours/week	0.3	714	0.4	797	-83	-588 to 423	0.75
Medication							
bDMARDs	75%	6410	72%	6276	134	-1403 to 1671	0.87
tsDMARDs	46%	1841	50%	2181	-341	-1539 to 858	0.58
csDMARDs	20%	62	18%	56	6	-39 to 51	0.79
NSAIDs	66%	148	57%	131	17	-32 to 65	0.50
Corticosteroids	30%	5	26%	5	0	-5 to 4	0.84
Total medication costs (SD)	92%	8465 (6314)	93%	8650 (6667)	-185	-2246 to 1877	0.86
TOTAL medical costs (SD)		15011 (9590)		14172 (10693)	838	-2404 to 4081	0.61
Average 1-year medical and non-medical costs per participant, by group.							
Non-medical costs							
Working hours, hours/week	8.1		10.0				
Absenteeism, hours/week	1.0		1.4				
Presenteeism, hours/week	2.1		2.9				
Productivity cost	-	3068	-	4,011	-944	-2734 to 846	0.30
Lost unpaid labor, hours/week	2.9	2547	3.2	2,850	-302	-1218 to 612	0.52
Household help, hours/week	0.4	324	0.3	233	92	-171 to 359	0.50
Informal care, hours/week	5.9	5279	4.9	4,387	892	-808 to 2593	0.30
Travel costs physiotherapy		94		25	70	59 to 80	< 0.001
Travel costs other healthcare		83		74	10	-13 to 32	0.41
TOTAL non-medical costs (SD)		11397 (11422)		11580 (9189)	-183	-3022 to 2656	0.90
TOTAL societal costs (SD)		26408 (15730)		25752 (13989)	657	-3748 to 5060	0.77

^# proportion of participants who had (intervention) therapy^

a The reported number of visits is here the average among participants with at least one visit.

b Other healthcare specialist: i.e., surgeon, gynecologist, pulmonologist, dermatologist, neurologist, ophthalmologist, and urologist.

c Other healthcare providers: i.e., psychologist, nurse other than rheumatology specialist nurse, medical podiatrist, speech therapist, skin therapist and practice assistant.

Abbreviations: bDMARDS, biological Disease-Modifying Antirheumatic Drugs; tsDMARD, targeted synthetic Disease-Modifying Antirheumatic Drugs; csDMARD conventional synthetic Disease-Modifying Antirheumatic Drugs; NSAIDs, Non-Steroidal Anti-Inflammatory Drugs.

### Other medical and non-medical costs

The difference in the total 1-year medical costs between the intervention and usual care groups was estimated at €838 (95% CI € -2404 to €4081, *p* = 0.61), favouring the usual care group (Table 3). Thus, the higher costs of physical therapy were in part compensated by lower costs associated with inpatient hospitalization and inpatient rehabilitation in the intervention group compared to the usual care group. Concerning the medication costs, the highest estimated expenses were associated with the use of biological (b) DMARDs and targeted synthetic (ts) DMARDs. While there were relatively similar proportions of participants using these specific types of DMARDs, the usual care group exhibited slightly higher costs of tsDMARDs (difference of €341 (95% CI €-1539 to €858), *p* = 0.58). Nevertheless, overall, there were no significant differences in the estimated medication costs between the groups.

Concerning the 1-year non-medical costs, the estimated difference between the intervention and usual care groups was €-183 (95% CI € -3022 to €2656, *p* = 0.90), favouring the intervention group, although the difference was not statistically significant. The small difference primarily arose from higher productivity costs in the usual care group (€944 (95% CI € -2734 to €846, *p* = 0.30)) and the higher costs of informal care in the intervention group (€892 (95% CI € -808 to €2593, *p* = 0.30)), essentially balancing each other out.

Consequently, the mean total societal costs per participant were €26408 (15730) for the intervention group and €25752 (13,989) for the usual care group, resulting in an overall difference of €657 (95% CI €-3748 to €5060) in favour of the usual care group. This overall difference was not statistically significant (*p* = 0.77).

### Cost-utility

**Fig. 2:**
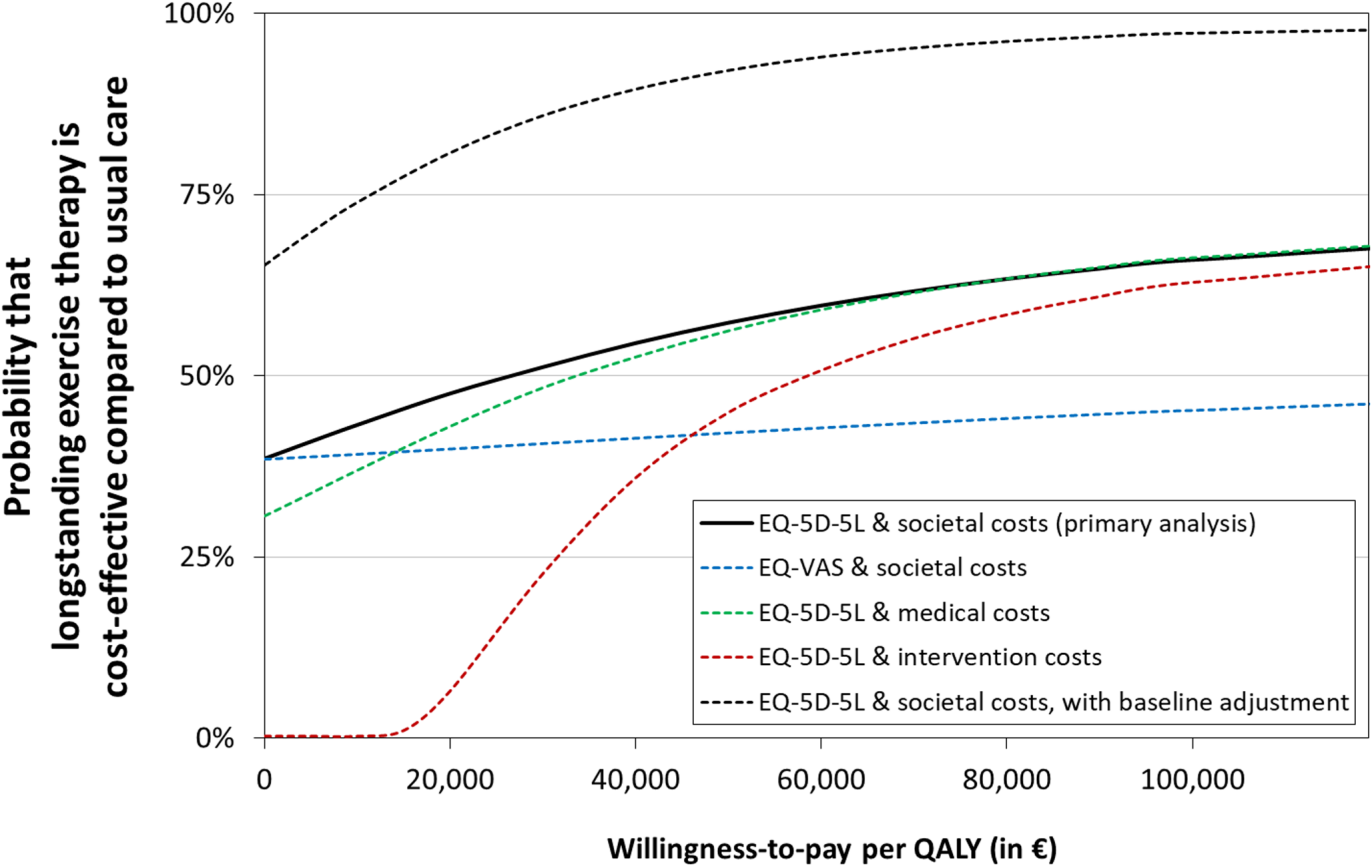
Cost-effectiveness acceptability curve showing the probability that longstanding exercise therapy is a cost-effective strategy compared to usual care over a range of values for the willingness-to-pay for an additional quality-adjusted life year (QALY). EQ-5D-5L, EuroQol-5 Dimensions-5 Levels; EQ-VAS, EuroQol Visual Analogue Scale

In the primary cost-utility analysis, the total societal costs were somewhat more favourable in the usual care group, whereas the QALYs based on the EQ-5D-5L were somewhat more favourable in the intervention group. However, none of these differences were statistically significant. As a result, when relatively low monetary values were assigned to QALYs, the probability that the intervention was cost-effective remained below 50% (as observed in the left segment of the acceptability curve in Fig. 2) and surpasses 50% when QALYs are valued higher (right segment in Fig. 2). For a willingness-to-pay threshold of 50,000 euro per QALY, which is considered appropriate for this patient population in the Netherlands, the intervention is estimated to have a 57% likelihood of being the cost-effective strategy. At 20,000 and 80,000 euro per QALY, this probability of cost-effectiveness was 48% and 63%, respectively. While the point estimate for the incremental cost-effectiveness ratio is a favourable €26.000 per QALY, this is an unstable estimate with an infinite confidence interval due to the non-significant difference in QALYs (95%CI 0 to infinity).

The sensitivity analysis using healthcare costs (instead of societal costs) was almost identical to the primary analysis using societal costs (ranging from 43 to 63%). For the narrow cost perspectives with only intervention costs and for the analysis with QALYs calculated from the EQ-VAS (instead of the EQ-5D-5L) the results were less favourable for the intervention (ranges 6%-58%, and 40%-44%, respectively). At baseline, compared to the usual care group, the intervention group had a lower average EQ-5D-5L (0.49 SD 0.27 versus 0.54 SD 0.24) and a relatively higher proportion of participants with higher education (47/109, 43.1% versus 31/104. 29/8%), although these differences did not reach statistical significance. Adjusting for these imbalances, the probability for the intervention to be more cost-effective than usual care increased, reaching 92% at the willingness-to-pay threshold of 50,000 euro per QALY (Fig. 2).

## Discussion

This RCT evaluated the societal costs and QALYs of 52-week long-term personalized exercise therapy intervention compared to usual care in individuals with axSpA and severe functional limitations. The total intervention costs were €1515 per participant. While the estimated total societal costs were €657 higher in the intervention group than in the usual care group, this difference did not achieve statistical significance (*p* = 0.77). The intervention group showed slightly more favourable health valuations, based on the EQ-5D-5 L, with only the difference at 26-weeks reaching statistical significance. The cost-utility analysis indicated higher costs in the intervention group yet with more favourable health outcomes. As a result, we found no clear economic preference: long-term exercise therapy need not be withheld for economic reasons. Sensitivity analyses supported the robustness of this finding.

This study fills a significant gap in the existing literature by offering insights into the cost-effectiveness of exercise therapy in individuals with axSpA and severe functional limitations, as literature addressing this specific topic is notably scarce. One previous economic analysis evaluated the cost-effectiveness of long-term supervised group exercise therapy versus individual home exercise therapy [13], another focused on exercise therapy as part of a comprehensive spa therapy program in addition to standard treatment, compared to standard treatment alone [14]. The results of these studies are somewhat comparable with the present study, as the costs of the long-term intervention were partially compensated by lower direct medical costs. However, several key distinctions set the two studies apart from our study. First, the study population differed. The participants in the study of Bakker et al. and van Tubergen et al. included only a subgroup of participants with AS and Bakker et al. only included individuals with AS who had no protheses of weight-bearing joints or serious comorbidities [13, 14]. In contrast, our study explicitly included participants with unstable medication, comorbidities or protheses. Second, the interventions of these studies differed, as they both involved a prolonged supervised group exercise therapy program lasting nine months, with a relatively fixed content and a predetermined number of sessions [13, 14], whereas the intervention in our study was tailored to meet the individual needs of the participants. Third, the control group in both studies had an exercise program; an unsupervised individual home exercise group and supervised group exercise therapy, respectively [13, 14]. In our study exercise was neither encouraged nor discouraged in the control group, and indeed 67% of the participants in the usual care group used physical therapy as part of their regular treatment. Considering these differences and the fact that the former studies were conducted over 20 years ago, a direct comparison with both studies seems inappropriate. The recent cost-utility analysis of the parallel RCT in patients with RA [11], with a similar population with regard to severe functional disability, may however be relevant for the interpretation of the current study results [15]. In that study, the difference in 1 year societal costs between the intervention and usual care groups was smaller than the observed difference in the current study, i.e. €180 (95% CI €-4493 to €4852, *p* = 0.94) versus €838 (95% CI € -2404 to €4081, *p* = 0.61). However, it must be noted that both in the parallel as the present study the the difference was not statistically significant. The effect with respect to QALYs was completely similar in both studies, with the mean difference according to the EQ-5D-5L being 0.02 (95% CI -0.05 to 0.09) in the RA study and 0.02 (95% CI -0.04 to 0.09) in the present study, again with both differences not reaching statistical significance. The economic analysis alongside an RCT in elderly individuals with complex mobility issues [16] showed cost savings (€848.80; 95% CI: -1607 to -90; *P* = 0.028) and greater improvements in QALYs (0.02; 95% CI: 0.00 to 0.03; *P* = 0.03). However, in that study all participants in the usual care group received physical therapy [16], whereas in the current study the decision to use physical therapy was at the discretion of the patient and/or clinicians, with only two-thirds of the patients actually using physical therapy. Consequently, the costs of physical therapy in our usual care group were notably lower than those in the study of de Vries et al., making it more challenging to demonstrate the cost-effectiveness of the intervention.

Thus, in line with the parallel RCT [15], in both that RCT and the current study, the total societal costs were higher in the intervention group than the usual care group, mostly due to the costs of the intervention. It could be hypothesized that providing this intervention might prevent the use of expensive forms of treatment, in particular admissions to a hospital or rehabilitation centre. However, as such admissions are relatively rare in this patient group, we did not have sufficient statistical power to demonstrate these differences. The power calculation for the present study was based on the clinical effectiveness rather than cost-utility [11, 12]. A study with a considerably larger sample size would be needed for that purpose. Similarly, it is conceivable that the intervention could have improved patients’ work ability through enhanced functional capacity [36]. However, we were unable to demonstrate any statistically significant difference in work ability in terms of presenteeism. In total, 34% of the participants from the intervention group and 38% from the usual care group had a paid job, making the sample size for a subgroup analysis too small. Moreover, our intervention did not specifically address work-related problems, so the impact on work may have been limited. But even if it would have addressed problems at work, the effect of a PT intervention focusing on that aspect is uncertain. Recently, a cost-effectiveness analysis of an RCT comparing a PT-led vocational intervention with usual care in people with RA and axSpA and work disability found that at 12 months, the intervention showed no significant clinical benefits, yet the QALYs were in favor of the intervention group by 0.05 (95% CI 0.00, 0.10) [37]. The mean intervention costs were €395 per participant (90% usage, mean 9.5 sessions). After 12 months, the societal costs were €4324 lower in the intervention group (95% CI €-8169, €-479), but that was mainly due to higher medication and presenteeism costs in the usual group rather than a decrease of these costs in the intervention group [37].

Our study has several strengths, such as a low attrition rate and low numbers of missing values on utilities and cost-related assessments. It remains to be established to what extent the results are generalizable to participants with axSpA and less severe functional limitations, who likely receive treatment less frequent and for a shorter duration. Another limitation is that the results may not be applicable to different healthcare systems outside the Netherlands. For instance, access to physical therapy may vary by country, influenced by variations in healthcare insurance systems. Moreover, the experimental segment of this study was limited to a 1-year time horizon. The exploration of long-term effects, particularly in the context of sustained exercise therapy usage, requires further investigation. Additionally, a limitation was the reliance on patient-reported data, which introduces potential differences of the actual use and costs of healthcare. The final limitation concerned the estimation of costs of physical therapy. Participants in the intervention group reported 30% more physical therapy sessions than reported by their PTs. This was partly due to intervention sessions that were not yet claimed for reimbursement by PTs, but also by physical therapy outside the intervention and overestimation by the participants. In the absence of a gold standard for the true use, resolving the discrepancy is challenging. Our analysis used the maximum of both sources, possibly overestimating the costs of physical therapy in the intervention group.

In summary, the results of this study revealed higher costs of longstanding exercise therapy, with the costs of the intervention being partly compensated by other savings and improved QALYs. Considering the improvement in clinical outcomes associated with the long-term exercise intervention compared to usual care [11], the Dutch Minister of Health, Welfare and Sport has decided to include the intervention for the particular subgroup of people with axSpA in the basic insurance from January 1, 2026 [https://www.rijksoverheid.nl/vraag-en-antwoord/zorgverzekering/veranderingen-basispakket; Accessed April 28, 2026]. However, additional research is necessary to validate the findings in larger groups and across diverse AxSpA populations and to investigate the long-term effects of the intervention.

## Data Availability

The data supporting this article will be made available upon reasonable request to the corresponding author.

## Declarations

### Prearchiving note

This report was not prearchived in a local repository.

### Previous publication

The results of this economic analysis have not been previously presented as an abstract.
